# Impact of Neoadjuvant Chemotherapy Administration Time of Day on Pathological Response in Patients with Early Triple-Negative Breast Cancer

**DOI:** 10.3390/cancers18081299

**Published:** 2026-04-20

**Authors:** Clémentine Bouchez, Simona Catozzi, Laetitia Someil, Caroline Cuvier, Léonor Drouin, Luis Teixeira, Catherine Miquel, Cédric De Bazelaire, Francis Levi, Jimmy Mullaert, Annabelle Ballesta, Sylvie Giacchetti

**Affiliations:** 1 Assistance Publique Hôpitaux de Paris (APHP), Saint Louis Hospital, Breast Diseases Unit, 75010 Paris, France; laetitia.someil@aphp.fr (L.S.); caroline.cuvier@aphp.fr (C.C.); leonor.drouin@aphp.fr (L.D.); luis.teixeira@aphp.fr (L.T.); 2 Université Paris Cité, Faculty of Medicine, 75005 Paris, France; 3 Cancer Systems Pharmacology, Inserm U1331, Institut Curie, PSL Research University, CBIO-Center for Computational Biology, Mines Paris, 75248 Saint-Cloud, France; simona.catozzi@curie.fr (S.C.); annabelle.ballesta@curie.fr (A.B.); 4 Université Paris Cité, Pathophysiology of Breast Cancer Team, HIPI, INSERM U976, 75005 Paris, France; 5 Saint Louis Hospital, Department of Pathology, APHP, 75010 Paris, France; catherine.miquel@aphp.fr; 6 Saint Louis Hospital, Department of Radiology, APHP, 75010 Paris, France; cedric.de-bazelaire@aphp.fr; 7 Inserm Unit 1193 HEPAREG, Faculty of Medicine, Paris-Saclay University, 94800 Villejuif, France; f.levi@warwick.ac.uk; 8 University of Versailles Saint-Quentin, Faculty of Medicine, 78646 Versailles, France; jimmy.mullaert@curie.fr; 9 Institute Curie, INSERM U1331 Computational Oncology Unit, Statistical Methods for Precision Medicine Lab, 92210 Saint-Cloud, France

**Keywords:** dose-dense, dose-intense neoadjuvant chemotherapy, administration time of day, chronotherapy, circadian drug timing, precision medicine, triple-negative breast cancer

## Abstract

**Background:** Triple-negative breast cancer (TNBC) is associated with poor prognosis, highlighting the need for innovative therapeutic strategies. Chronomodulated chemotherapy yielded improved efficacy and tolerability in several malignancies; however, evidence in breast cancer remains limited. This study investigated the association between the time of day of administration (ToDA) of neoadjuvant chemotherapy (NAC) and clinical outcomes in patients with TNBC treated in a day hospital setting. **Methods:** This retrospective cohort included patients treated at Saint-Louis Hospital with neoadjuvant dose-dense, dose-intense cyclophosphamide–epirubicin followed by weekly paclitaxel. Infusion start times of each chemotherapy agent were systematically recorded. The primary endpoint was pathological complete response (pCR; Residual Cancer Burden [RCB] = 0). Secondary endpoints included RCB classes, early metabolic response, treatment tolerance, and 36-month event-free survival (EFS). Patients were classified into early or late infusion groups using multiple ToDA cut-offs, and cosine-based analyses were performed. **Results:** Ninety-four patients (median age 51 years) received NAC between 9:00 and 17:40 which aligns with the day hospital unit’s opening hours. With a median follow-up of 39.9 months, the pCR rate was 48.9%. No significant differences were observed between early and late infusion groups for any endpoint, regardless of ToDA cut-off or analytical approach. **Conclusions:** This first chronotherapeutic study in patients with early TNBC showed no association between ToDA of NAC administered between 9:00 and 17:40 and histological response or EFS. Although limited by sample size and the restricted infusion time window, our study provides novel data and methodological insights supporting further investigation of chronotherapy in patients with breast cancer.

## Simple Summary

While chronotherapy has demonstrated improved tolerability and efficacy in several malignancies, its impact on breast cancer remains unclear. This retrospective study of 94 patients treated at Saint-Louis Hospital (France) investigated the effect of the time of day of administration (ToDA) of neoadjuvant epirubicin–cyclophosphamide followed by paclitaxel on outcomes in patients with triple-negative breast cancer (TNBC). Within the investigated time window (09:00–17:40), we found no association between chemotherapy timing and histological response or event-free survival. Apart from being the first study to examine ToDA of NAC in TNBC, key strengths of our study include a homogeneous population, rigorous recording of chemotherapy ToDA, and innovative methodology to determine the timing cut-off distinguishing early and late groups. Although limited by sample size and constrained timing coverage of approximately one third of the circadian period, our study provides novel and methodologically rigorous data that contribute to the evaluation of chronotherapy in breast cancer.

## 1. Introduction

Triple-negative breast cancer (TNBC) is defined by the absence or low expression of the estrogen receptor (ER) and progesterone receptor (PR), as determined by immunohistochemistry in fewer than 10% of tumor cells [1], together with the lack of human epidermal growth factor receptor 2 (HER2) overexpression or gene amplification [2,3,4]. Patients with TNBC have poorer breast cancer-specific survival and overall survival (OS) compared with those with hormone receptor positive and/or HER2-overexpressing tumors [5].

Despite this poor prognosis, TNBC is highly sensitive to chemotherapy and neoadjuvant therapy has progressively become the standard of care for early-stage disease particularly with dose-dense regimens. At our institution, patients with TNBC were treated with a dose-dense, dose-intense (DD-DI) NAC regimen, also called the “SIM” regimen for “Sein Inflammatoire Métastatique” [6]. This protocol consists of four cycles of epirubicin (75 mg/m^2^) combined with cyclophosphamide (1200 mg/m^2^) every two weeks (q2w) followed by 12 weekly infusions (qw) of paclitaxel (80 g/m^2^). This regimen has demonstrated a significant improvement in pathologic complete response (pCR) rates and in long-term disease-free survival compared with conventional sequential association of cyclophosphamide and anthracycline, followed by taxanes (EC-T) [7,8]. Moreover, achieving pCR after NAC is strongly associated with favorable survival outcomes in patients with TNBC, and represents a relevant surrogate endpoint [9,10,11].

Growing evidence suggests that circadian biology impacts the efficacy and toxicity of anticancer treatments [12]. Following the 2017 Nobel Prize in Physiology or Medicine awarded to Hall, Rosbash, and Young for the discovery of the molecular circadian clock, the interest in chronotherapy—i.e., the administration of treatments according to biological rhythms—has been renewed in the scientific community. Time-of-day dependencies in drug efficacy and toxicity have been documented for more than 50 anticancer compounds in preclinical studies [13,14] which may be explained by chronopharmacological or chronopharmacodynamic effects [15]. Several randomized trials in patients with cancer have demonstrated improved tolerability in chronotherapy arms compared with conventional, non-time-specified administration, while maintaining or even improving response rates and/or survival outcomes. A systematic review of 18 randomized trials in patients with six cancer types highlighted a significant decrease in toxicity incidence in 77% of the trials, with similar or improved efficacy [16]. These findings support further investigations regarding the ToDA chemotherapy administration in patients with cancer. Importantly, the optimal ToDA for anticancer drugs may vary according to patients’ characteristics, including sex and age [17,18,19]. Ongoing research aims to elucidate the molecular and physiological mechanisms underlying inter-patient variability in circadian drug responses. An important study in this area leveraged data from the Genotype-Tissue Expression (GTEx) project, composed of 16,000 transcripts across 46 tissues from 914 donors, to infer each donor’s circadian phase and estimate age- and sex-specific circadian profiles of gene expression [20]. Clock transcripts showed conserved timing relationships and tight synchrony throughout the body, while rhythm amplitude decreased with aging. Notably, women exhibited stronger circadian oscillations in genes involved in drug metabolism (e.g., CYP1A1, CYP1B1, CYP3A4), detoxification (e.g., GSTA1), and transport (e.g., ABCB1, ABCC1, ABCC2) [20,21].

Given the scarcity of data on the influence of ToDA on treatment response and the survival of patients with breast cancers [22], we conducted a retrospective cohort study to evaluate the association between the timing of DD-DI NAC administration and tumor response, as well as event-free survival, in patients with early TNBC.

## 2. Patients and Methods

### 2.1. Cohort Population

Patients treated at the Breast Cancer Unit of Saint-Louis Hospital (Paris, France) for primary TNBC (ER and PR < 10% and no Her-2 overexpression, i.e., 0, 1+, 2+ with negative in situ hybridization) with neoadjuvant DD-DI chemotherapy were included. At baseline evaluation, all patients underwent a core needle breast biopsy and, when indicated, axillary lymph node biopsy, in addition to a PET-scan. BRCA status was assessed on tumor tissue to identify somatic BRCA mutations, following patient consent.

### 2.2. Chemotherapy and ToDA Assessment

Patients received a DD-DI NAC regimen consisting of four cycles of epirubicin (75 mg/m^2^) and cyclophosphamide (1200 mg/m^2^) administered every two weeks (SIM regimen) [6] followed by 12 weekly injections of paclitaxel (80 mg/m^2^). The exact start time of each anticancer agent infusion was prospectively recorded at the patient’s bedside by trained nursing staff. Efforts were made by the nursing staff to ensure accurate documentation of the infusion start times for each anticancer treatment. Emphasis was placed on reliable ToDA assessment and raising awareness among the team about the importance of precisely reporting the beginning of every treatment. Following NAC, patients underwent either lumpectomy or total mastectomy with lymph node surgery. Adjuvant chemotherapy with capecitabine was proposed for those patients without pCR and radiotherapy was administered whenever indicated.

### 2.3. Outcomes

The primary outcome of the study was to determine the pathological complete response (pCR) rate, defined as the absence of any residual invasive tumor in the breast and lymph nodes at the time of definitive surgery [9,10,11] according to the ToDA. The RCB values were computed using a formula based on five variables (the primary tumor bed area, overall cancer cellularity, percentage of in situ carcinoma, number of positive lymph nodes and the diameter of the largest lymph node metastasis) using the Residual Cancer Burden Calculator (https://www3.mdanderson.org/app/medcalc/index.cfm?pagename=jsconvert3, assessed on 15 April 2026), as described by Symmans et al. [23]. The RCB was analyzed as classes, 0 indicating pathological complete response (pCR). The classes of RCB 1, 2 or 3 were defined using two empirically derived cut-off points selected as the 87th percentile of a reference study (RCB 3.28) for separating RCB 3 from RCB 1–2 and the 40th percentile of the same investigation (RCB 1.36) for discriminating RCB 1 from RCB 2 [23,24].

Secondary endpoints were: (i) early metabolic response after two courses of NAC, specifically assessing breast maximum standardized uptake value (SUV_max_) variation (∆SUV_max_ = (SUV2-SUV1) × 100/SUV1 (%); SUV1 corresponding to breast SUV_max_ on initial PET-scan, and SUV2 corresponding to breast SUV_max_ on PET-scan after two courses of NAC); (ii) the percentage of chemotherapy dose reductions and treatment delays by ≥7 days; (iii) the event-free survival (EFS) over a 36-month period [25] defined as the time between the date of diagnosis and the date of progression, death of any cause or date of last news; and (iv) individual parameters of the RCB score (tumor bed surface area, tumor cellularity, histological lymph node involvement pN).

### 2.4. Selecting Infusion ToDA Cut-Offs

SIM and paclitaxel regimens were analyzed separately. For each chemotherapy, patients were classified into early and late groups based on their median infusion times. Such a metric was chosen as it allows both cut-off-based discrete analysis and continuous analysis, as described hereafter. For population dichotomization, three strategies were used to select the early/late cut-off. First, the timing cut-off was set as the median of median infusion times among patients. Second, the optimized cut-off was selected to maximize the difference in RCB between the early and late patient groups, using the Wilcoxon rank test applied to RCB categories (i.e., 0, 1, 2 or 3). A third approach aimed to optimize the cut-off time by maximizing the difference in restricted mean survival time (RMST) between the early and late groups [26]. RMST was determined by evaluating the areas under the event-free survival (EFS) curves up to the time point when 90% of events had occurred. For the second and third approaches, the optimal cut-off was obtained by a grid search over possible timing values. In both cases, the selected cut-off satisfied the constraint that the smallest resulting group comprised at least 20% of the study population.

### 2.5. Statistical Analysis

For continuous variables, mean/SD or median/interquartile range were reported based on the normality assumption (Shapiro–Wilk test). Categorical variables were presented as absolute values and proportions. Patient group differences were assessed using either the *t*-test or Kruskal–Wallis test for normally or non-normally distributed continuous variables respectively, and by Pearson’s chi2 test or Fisher’s exact test for categorical variables. The Wilcoxon rank test was used for ordered categorical variables.

The impact of timing groups on pCR (RCB 0 vs. RCB 1,2,3) was further investigated using logistic regression for the three investigated timing cut-off values. A power calculation based on the hypothesis of a binary exposure (i.e., early or late timing of chemotherapy) shows that this test of pCR based on logistic regression, with our sample size of 94 patients and an alpha risk of 5%, has 80% power to detect an association with OR = 1.8, if the exposure is strictly balanced (i.e., half of the patients in each timing group). In the case of an imbalanced exposure with 20% of observations in one group and 80% in the other, and with the same sample size, the detectable effect size with 80% power is OR = 2.1. To evaluate the impact of intra-patient ToDA variability, weighted logistic regression was additionally conducted by assigning weights equal to the inverse of each patient’s variance of infusion timing over cycles (with an increment of one added to prevent division by zero). The infusion timing dependency of individual parameters of the RCB score (tumor bed surface area, tumor cellularity, histological lymph node involvement pN) was analyzed by the method of the cosinor, considering the timing variable as continuous, and using either SIM or paclitaxel median infusion timing for each patient [27]. EFS was measured starting from the date of TNBC diagnosis. Events included invasive local, regional, or metastatic relapse, contralateral breast cancer, or death from any cause, whichever occurred first. It was estimated using Kaplan–Meier curves, with differences between patient group survival assessed through a log-rank test. Univariable Cox models were employed to gauge the impact of chemotherapy early/late timing group [2]. The 24 h periodic risk model is characterized by two parameters: an acrophase (time of peak) and an amplitude (half-distance between minimum and maximum). In addition, a spline-based Cox model was also applied to EFS data, and the degree of freedom was optimized using the AIC criteria. Subsequently, a sensitivity analysis examining intra-patient ToDA variability was conducted by running the cosine-based and spline analyses enforcing weights set to the inverse of each patient’s variance of infusion timing over all cycles they received (with an increment of one added to prevent division by zero).

## 3. Results

### 3.1. Baseline Characteristics of the Overall Population

Between January 2018 and May 2022, 94 patients received DD-DI neoadjuvant chemotherapy (NAC) with the SIM–paclitaxel regimen for non-metastatic TNBC at the Breast Cancer Unit of Saint-Louis Hospital. The median age at diagnosis was 51 years (IQR [41–63], Table 1). Forty-eight patients (51%) were pre-menopausal. More than 75% of the patients had a performance status of 0 or 1. The median body mass index (BMI) was 25.9 kg/m^2^ (IQR [21.7, 28.7]). Most patients had T3 or T4 tumors (57 patients, 60.6% of the whole population). Most tumors were grade III, and the median proliferation index (Ki67) was 75% (IQR [60, 90]). Axillary lymph node involvement was clinically suspected in 42 patients (44.7%) and histologically proven in 29 of these 42 patients (69%). BRCA1 and BRCA2 somatic mutations were analyzed in 75 tumor samples (79.7%) and were detected respectively in 11 and 2 of them (13/75, 17.33%) (Table 1).

### 3.2. Treatment Duration, Response and Event-Free Survival in the Whole Population

Among the 94 patients, 89 (94.7%) received the four courses of DD-DI epirubicin–cyclophosphamide and 87 (92.5%) received at least nine weekly injections of paclitaxel (Supplementary Table S1). Ninety-two patients (98%) underwent breast and axilla surgery. Forty-five patients (48.9%) achieved pCR whereas residual triple-negative invasive adenocarcinoma was found in breast and/or in lymph nodes of 49 patients (52.1%). The mean early metabolic response was −55.7% (SD 24.1%). A dose reduction in chemotherapy was implemented in 29.8% of the population and almost half of the population (47.9%) had at least one course of chemotherapy delayed by a minimum of 7 days.

The median follow up was 39.9 months (IQR: [29.2–47.8]). Within the study, one patient had a local recurrence, 12 patients developed metastatic recurrence, and five patients died. Event-free survival at 36 months was 84% (95% CI: 77–93%) (Table 2).

### 3.3. Distribution of ToDA of NAC

**Figure 1:**
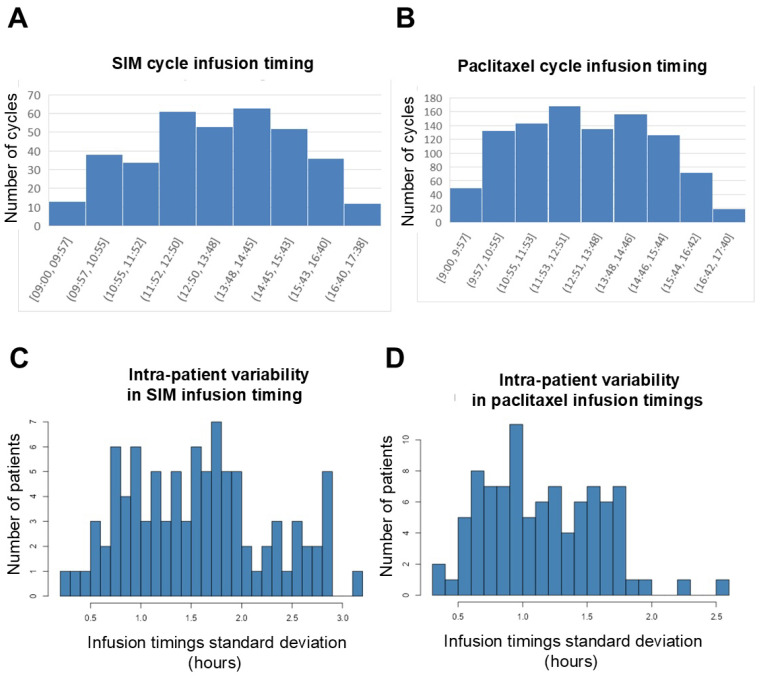
(**A**,**B**) Distribution of time-of-day individual infusions of SIM (**A**) and paclitaxel (**B**) for all patients. (**C**,**D**) Intra-patient variation in infusion timing. Standard deviations of infusion timing of SIM (**C**) or paclitaxel (**D**) for each patient.

The ToDA infusion of SIM and paclitaxel was unavailable for 8 out of 370 and 39 out of 1042 injections respectively (corresponding to 2% and 3.7% of missing values). All chemotherapy infusions were administered between 9:00 and 17:40 which aligns with the day hospital unit’s opening hours (Figure 1A,B). The median of patients’ median infusion times was 13:38 for SIM and 12:55 for paclitaxel. Intra-patient variability in infusion timing during the entire neoadjuvant treatment was moderate as patients’ median ToDA standard deviation was ±1 h 34 for SIM and ±1 h 07 for paclitaxel (Figure 1C,D). Using the median times to dichotomize the exposure, we found that thirty-six (38.3%) and thirty-five patients (37.2%) belonged to the early or late groups for both SIM and paclitaxel respectively (concordant groups). Conversely, twelve patients (12.8%) had most SIM injections in the early and paclitaxel in the late group. Eleven patients (11.7%) had the reverse pattern (discordant groups).

### 3.4. Association Between SIM ToDA and Outcomes

In the first early/late dichotomization method, we selected the median of patients’ median infusion times as the ToDA cut-off value. For SIM, this cut-off was at 13:38 and resulted in two equivalent patient groups with comparable clinical and tumor characteristics (Supplementary Table S2), except for (i) the proliferation index which was significantly higher in the late group compared to the early group (median [Q1, Q3]: 80% [70%, 90%] vs. 70% [45%, 80%]; *p* = 0.02), and (ii) the tumor metabolic activity which was slightly higher in the late group compared to the early group (SUVmax: 14.95 [11.80, 21.23] vs. 12.60 [8.97, 17.08]; *p* = 0.02) (Supplementary Table S2).

**Figure 2:**
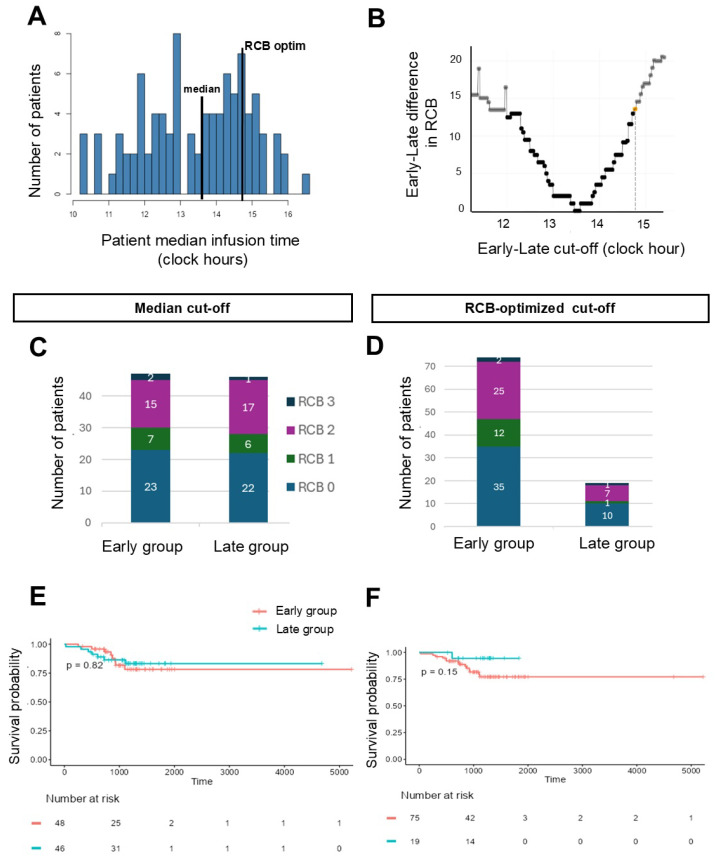
**Association between SIM ToDA and treatment outcomes for various early/late cut-off values.** (**A**) Distribution of patients’ median SIM infusion timing. Black lines indicate the three investigated early/late cut-off values: (i) the median of patients’ median timing, (ii) the cut-off maximizing the early/late difference in RCB, (iii) the cut-off maximizing the early/late differences in EFS (see Methods). (**B**) Early/late difference in RCB (displacement of Wilcoxon rank test) with respect to tested timing cut-off values. The dotted line indicates the optimal cut-off value corresponding to the maximum difference under group size restrictions. (**C**,**D**) Number of patients in the early and late groups in each RCB class using either the median or RCB-optimized cut-off for defining patient groups. (**E**,**F**) Kaplan–Meier curve of EFS using either the median (**E**) or RCB-optimized (**F**) cut-off value to define early and late patient groups. p values refer to log-rank test.

The rate of pCR and the distribution of patients according to histological response were comparable between both groups (logistic regression comparing RCB 0 vs. 1,2,3, *p* = 0.91; RCB classes proportions, *p* = 0.9, Figure 2, Table 3). Accounting for intra-patient variability in the ToDA of all cycles yielded similar results (weighted logistic regression, *p* = 0.94). The proportions of patients achieving pCR (RCB 0) were 48.9% for the early group and 47.8% for the late group. In the early group, 14.9% of patients had an RCB of 1, 31.9% an RCB of 2 and 4.3% an RCB of 3. In the late group, RCB 1 was observed in 13% of patients, RCB 2 in 37% and RCB 3 in 2.2% (Table 3). Early metabolic responses were similar across timing groups (*p* = 0.68, Table 3). No significant differences were observed between groups in the occurrence of either dose reduction or chemotherapy delay (*p* = 0.09 and *p* = 0.28 respectively, Table 3). EFS did not differ significantly between early and late groups (log-rank *p* = 0.82, univariable Cox HR (95% CI) = 0.89 (0.32, 2.45), *p* = 0.8, Figure 2). Likewise, the proportion of patients alive at 3 years did not vary significantly according to SIM timing (Table 3).

We next optimized the ToDA cut-off value by maximizing the difference in either RCB class proportions (Figure 2, Table 3) or EFS (Supplementary Figure S1, Supplementary Table S3) between the early and late patient groups. RCB- or EFS-optimized cut-off values for SIM timing were very close and equal to 14:48 and 14:43, respectively. These cut-off values yielded early and late populations with similar characteristics including the proliferation index Ki67 (Supplementary Table S2). With the RCB-based cut-off, both the pCR rate and the proportions of RCB scores were similar between early and late groups (logistic regression *p* = 0.75 and Wilcoxon rank test *p* = 0.629, respectively). Accounting for intra-patient variability in injections’ ToDA yielded similar results (weighted logistic regression *p* = 0.76). The proportion of patients achieving pCR was 47.3% for the early group (before 14:48) and 52.6% for the late group (after 14:48). In the early group, 16.2% of patients had an RCB of 1, 33.8% an RCB of 2 and 2.7% an RCB of 3. In the late group, RCB 1 was observed in 5.3% of patients, RCB 2 in 36.8% and RCB 3 in 5.3% (Table 3). With the EFS-optimized cut-off, the proportion of patients achieving a pCR was 48.6% for the early group and 47.6% for the late group (logistic regression comparing RCB 0 vs. 1,2,3 *p* = 0.94; weighted logistic regression to test intra-patient ToDA variability *p* = 0.88; RCB class proportions: *p* = 0.976, Supplementary Table S3). Regardless of the method, there were no statistically significant differences between early and late patient groups for early metabolic response, dose reduction, delayed chemotherapy cycles or for EFS (RCB-based cut-off, log-rank *p* = 0.15; Cox univariate *p* = 0.1, HR(95% CI) = 0.42 (0.15, 1.19) Figure 2, Table 3; EFS-based cut-off, log-rank *p* = 0.11; Cox univariate *p* = 0.15, HR (95% CI) = 0.22 (0.029, 1.697), Supplementary Figure S1, Supplementary Table S3). Likewise, individual parameters of RCB score (tumor bed surface area, tumor cellularity, histological lymph node involvement pN) did not significantly differ depending on ToDA of SIM (Supplementary Figure S2).

To rule out possible artifacts of the patient population dichotomization process, we next considered the ToDA variable as continuous and tested the impact of timing using cosine-based approaches. The timing dependency of the RCB score and of its individual parameters (tumor bed surface area, tumor cellularity, histological lymph node involvement pN), displayed no statistically significant association with SIM infusion timing (cosinor *p* > 0.05, Supplementary Figure S1). In addition, the cosine-based Cox model analysis concluded there was an absence of sinusoidal association between SIM timing and EFS, both with and without consideration of intra-patient ToDA variability through patient weighting (null amplitude significance *p* > 0.1). The spline-based analysis found that the linear model best fits the data, but results were not significant with any methods (slope: −1, *p* = 0.12 or *p* = 0.3 without or with patient weighting respectively).

### 3.5. Association Between Paclitaxel ToDA and Outcomes

**Figure 3:**
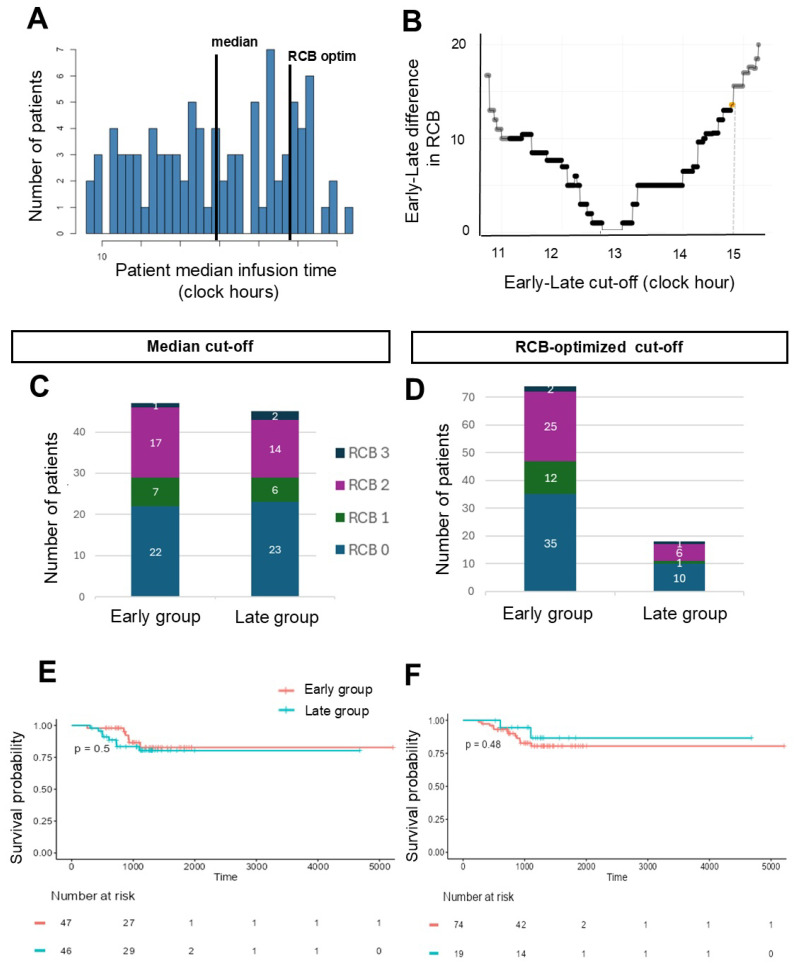
**Association between paclitaxel ToDA and treatment outcomes for various early/late cut-off values.** (**A**) Distribution of patients’ median SIM infusion timing. Black lines indicate the three investigated early/late cut-off values: (i) the median of patients’ median timing, (ii) the cut-off maximizing the early/late difference in RCB, (iii) the cut-off maximizing the early/late differences in EFS (see Methods). (**B**) Early/late difference in RCB (displacement of Wilcoxon rank test) with respect to tested timing cut-off values. The yellow dot indicates the optimal cut-off value corresponding to the maximum difference under group size restrictions. (**C**,**D**) Number of patients in the early and late groups in each RCB class using either the median or RCB-optimized cut-off for defining patient groups. (**E**,**F**) Kaplan–Meier curve of EFS using either the median (**E**) or RCB-optimized (**F**) cut-off value to define early and late patient groups. p values refer to log-rank test.

We then dichotomized the cohort according to the median of paclitaxel patients’ median times, i.e., 12:55, which yielded two equivalent patient groups with well-balanced clinical and tumor characteristics (Supplementary Table S4), except for the tumor proliferation index which was higher in the late group (early vs. late median [Q1, Q3] Ki67 index: 70% [50, 80] vs. 80% [70, 90], *p* = 0.02). A comparable rate of pCR was observed across timing groups (46.8% in the early group, 51.1% in the late group, logistic regression *p* = 0.68, weighted logistic regression accounting for intra-patient ToDA variability *p* = 0.82), as well as a similar distribution of the different RCB classes (*p* = 0.88, Table 4) and early metabolic response (*p* = 0.85, Table 4). The occurrence of chemotherapy dose reduction or delay was not statistically different between both timing groups (*p* = 0.69 and 0.26 respectively, Table 4). EFS of early and late groups was not significantly different (log-rank *p* = 0.82, univariable Cox *p* = 0.5, HR (95% CI) = 1.437 (0.5, 4.15), Figure 3). Three-year EFS was also comparable (*p* = 0.45, Table 4).

We next optimized the dichotomization cut-off by maximizing early vs. late differences in either RCB class proportion (Figure 3, Table 4) or EFS (Supplementary Figure S3, Supplementary Table S5). The RCB- or EFS-optimized cut-off values for paclitaxel timing were close and equal to 14:48 and 14:15, respectively, and yielded comparable early and late patient groups with differences only in the Ki67 proliferation index (Supplementary Table S4). ToDA dependencies were not found in the pCR rate (for RCB-based cut-off, logistic regression *p* = 0.79, patient ToDA variability weighted logistic regression *p* = 0.84, Figure 3, Table 4; for EFS-based cut-off, logistic regression *p* = 0.55, weighted logistic regression *p* = 0.68, Supplementary Figure S3, Supplementary Table S5), histological response (RCB classes), tumor surface area, tumor cellularity, histological lymph node involvement (Supplementary Figure S4), early metabolic rate nor in the occurrence of dose reduction or chemotherapy delay. EFS was also similar for both timing groups (for RCB-optimized cut-off: log-rank *p* = 0.48; Cox univariate *p* = 0.71, HR (95% CI) = 1.22 (0.82, 3.52); EFS-optimized based cut-off, log-rank *p* = 0.21; Cox univariate *p* = 0.23, HR (95% CI) = 0.4 (0.09, 1.78), Figure 3). Consistent with these findings, the timing dependency of the RCB score and of its individual parameters (tumor bed surface area, tumor cellularity, histological lymph node involvement pN) displayed no statistically significant association with SIM infusion timing, when considered as a continuous variable (cosinor *p* > 0.05, Supplementary Figure S2). Similarly, sinusoidal Cox model analysis concluded there was an absence of association between paclitaxel timing and EFS whether or not intra-patient ToDA variability was considered through patient weighting (null amplitude significance *p* > 0.1). Additionally, the spline-based ToDA univariate model was non-significant (optimal degree, 3.02; *p* = 0.3) with any methods (*p* = 0.3 or *p* = 0.7 without or with patient weighting, respectively).

## 4. Discussion

In this cohort of patients with early TNBC, we retrospectively investigated the association between ToDA of DD-DI chemotherapy and patients’ outcomes for chemotherapy infusion courses initiated between 09:00 and 17:40. Within such a limited time window, we found no statistically significant differences in histological responses or EFS according to ToDA of either SIM or paclitaxel.

This study has several strengths. To our knowledge, it is the first to investigate the impact of timing of dose-dense, dose-intense neoadjuvant chemotherapy (DD-DI NAC) on outcomes in patients with TNBC. Chemotherapy administration timing was rigorously recorded, as nurses were extensively trained to report the exact timing of infusions at the patient’s bedside. The study population was relatively homogeneous, comprising patients with non-metastatic TNBC in good general condition; most had a performance status of 0. Furthermore, whereas most chronotherapy studies have focused on the impact of treatment timing on toxicity, we chose to evaluate the effect of ToDA of NAC on treatment efficacy using the validated endpoint of pCR. Assessment of chemotherapy tolerance is challenging in a retrospective setting due to incomplete toxicity reporting. Therefore, the proportion of chemotherapy dose reductions and treatment delays were used as surrogate markers of treatment tolerance.

A key aspect addressed in this study is the methodology for analyzing TODA impact on clinical outcomes. For population dichotomization, we used both the median of the patients’ median infusion time and outcome-optimized thresholds based on RCB and EFS. The optimized cut-off times were remarkably consistent across the evaluated endpoints: for SIM, 14:48 for RCB and 14:43 for EFS; for paclitaxel, they were 14:48 for RCB and 14:15 for EFS. Baseline characteristics were highly comparable between early and late groups as the only difference observed was the Ki67 proliferation index; however, this variable is not clinically relevant in early TNBC, as proliferation is almost always high (median [Q1–Q3], 75% [60–90]). Thus, such similarity of both timing groups allowed for an analysis of the timing effect with limited confounding factors. Next, we further complemented these cut-off-based discrete approaches with cosine-based or spline-based analysis, acknowledging the fact that ToDA is a continuous periodic variable, to unravel any artifact linked to patient population dichotomization.

This study has also several limitations. First, this is a single-center retrospective study, the sample size was limited, and the infusion timing explored was restricted to daytime hospital hours, covering only approximately one third of the 24 h period. Therefore, our conclusion regarding the lack of association between NAC timing and efficacy does not exclude the possibility of time-dependent effects, which would require evaluation across the entire circadian cycle.

Preclinical studies in mice have demonstrated the impact of treatment timing on both the toxicity and antitumor activity of various drugs used in breast cancer. Scheving et al. reported a greater efficacy of a cyclophosphamide–doxorubicin regimen in male mice bearing advanced L1210 leukemia when administered at ZT13 (Zeitgeber Time; 1 h into active phase), compared with ZT1 (1 h into rest phase) [28]. Borniger et al. further investigated mechanisms underlying time of day-dependent effects in murine breast cancer models hypothesizing that the ToDA may alter inflammatory responses to chemotherapeutics [29]. To test this hypothesis, they administered cyclophosphamide and doxorubicin (Cyclo/Dox), to female mice near the beginning of the rest phase or of the active phase. Mice treated during the rest phase exhibited greater body weight loss and increased expression of pro-inflammatory genes in the spleen compared with those treated during the active phase, suggesting time- and tissue-specific immune responses to chemotherapy. Granda et al. further demonstrated large dosing time dependencies in the tolerability and the efficacy of single-agent doxorubicin and docetaxel or in combination in healthy and mammary adenocarcinoma MA13/C-bearing male mice [30]. Docetaxel showed optimal tolerability and efficacy when administered near the middle of the rest phase, while a similar trend was observed for doxorubicin, although without statistical significance. Rodent studies indicate that the optimal timing for administering anthracyclines, cyclophosphamide, and docetaxel occurs during the middle of the rest phase or at the beginning of the active phase (ZT 7–14), corresponding in humans to the middle of the night to early morning. In a recent review on anthracycline cardiotoxicity according to the ToDA of anthracycline in rodents and humans, the authors concluded that chronomodulated anthracycline administration could reduce cardiotoxicity but that it remains premature at this stage to define precise timing recommendations [31]. Nevertheless, extrapolation from preclinical data suggests that administration in the middle of the night or early morning may represent a potentially optimal window, which was not documented in this day hospital study.

Consequently, although we did not observe an association between the ToDA of neoadjuvant chemotherapy (NAC) and treatment efficacy within this time window, our findings cannot definitively exclude the presence of time-dependent effects. By studying the timing of anticancer drug administration, treatment efficacy could be maximized while minimizing adverse effects. This approach should be mandatory in the development of new drugs.

## 5. Conclusions

In this single-center retrospective cohort of 94 patients with TNBC, we present the first clinical study evaluating the association between ToDA of DD-DI neoadjuvant chemotherapy and clinical outcomes. Within the documented time window restricted to daytime administration (9:00–17:40), we found no evidence that ToDA influenced pathological response or event-free survival in patients with TNBC. Although limited by sample size and constrained circadian coverage, our study provides novel, methodologically rigorous data that contribute to the clinical evaluation of chronotherapy in breast cancer.

## Figures and Tables

**Table 1:** Characteristics of overall population.

	n = 94
Age at diagnosis (years old), median [Q1, Q3]	51.39 [41.39, 63.19]
Performance status at diagnosis, n (%)	
- 0	68 (72.3)
- 1	4 (4.3)
- Missing	22 (23.4)
Body mass index (kg/m^2^), median [Q1, Q3]	25.95 [21.70, 28.70]
Parity, n (%)	
- Nulliparity	24 (25.5)
- Multiparity	70 (74.5)
Menopausal status, n (%)	
- Non-menopausal	48 (51.1)
- Menopausal	46 (48.9)
Tumor size (according to TNM/AJCC 2018), n (%)	
- T1–T2	37 (39.4)
- T3–T4	57 (60.6)
Grade (Elston & Ellis), n (%)	
- II	11 (11.7)
- III	83 (88.3)
Proliferation index Ki67 (%), median [Q1, Q3]	75 [60, 90]
Tumor-infiltrating lymphocytes (%), median [Q1, Q3]	10.0 [5, 30]
Breast tumor metabolic activity on TEP-FDG scan (SUVmax), median [Q1, Q3]	13.80 [10.05, 18.67]
*BRCA* somatic mutation, n (%)	
- *BRCA1*	11 (11.7)
- *BRCA2*	2 (2.1)
- Absent	61 (64.9)
- Missing	20 (21.3)
Clinical or radiological lymph node invasion, n (%)	
- Present	42 (44.7)
- Absent	52 (55.3)
Proven histological lymph node invasion, n (%)	29 (30.8)

For continuous variables, mean/SD or median/interquartile range were reported based on the normality assumption (Shapiro–Wilk test). Categorical variables were presented as absolute values and proportions. Patient group differences were assessed using either the *t*-test or Kruskal–Wallis test for normally or non-normally distributed continuous variables respectively, and by Pearson’s chi2 test or Fisher’s exact test for categorical variables. The Wilcoxon rank test was used for ordered categorical variables. Abbreviation: n, number of patients.

**Table 2:** Treatment duration, response and event-free survival in the overall population.

	n = 94
Residual Cancer Burden (class), n (%)	
0	45 (48.9)
I	14 (14.9)
II	32 (34.0)
III	2 (2.1)
Missing but no pCR	1 (1.1)
∆SUVmax (%), mean (SD)	−55.7 (24.1)
Dose reduction in chemotherapy, n (%)	28 (29.8)
Of SIM regimen	7 (7.4)
Of paclitaxel regimen	21 (22.3)
Delayed chemotherapy ≥ 7 days, n (%)	45 (47.9)
Of SIM regimen	18 (19.1)
Of paclitaxel regimen	27 (28.7)
Recurrence, n (%)	
Local	1 (1.1)
Locoregional	0 (0.0)
Contralateral	2 (2.1)
Metastatic	12 (12.8)
Number of deaths, n (%)	5 (5.3)
Event-free survival, (%, 95CI)	
At 12 months	97% (93–100)
At 24 months	90% (84–96)
At 36 months	84% (77, 93)

For continuous variables, mean/SD or median/interquartile range were reported based on the normality assumption (Shapiro–Wilk test). Categorical variables were presented as absolute values and proportions. Patient group differences were assessed using either the *t*-test or Kruskal–Wallis test for normally or non-normally distributed continuous variables respectively, and by Pearson’s chi2 test or Fisher’s exact test for categorical variables. The Wilcoxon rank test was used for ordered categorical variables. Differences in the survival of groups are assessed with a log-rank test. Abbreviations: n, number of patients; RCB, residual tumor burden.

**Table 3:** Association between SIM ToDA and outcomes.

Chemotherapy Regimen		SIM					
	Missing (%)	Median Infusion Time (13:38)			Optimizing Cut-Off by Maximizing Differences in RCB Classes (14:48)		
		Early	Late	*p*-Value	Early	Late	*p*-Value
**Number of patients**		48	46		75	19	
**RCB (class), n (%)**	1.1			0.908			0.629
**0**		23 (48.9)	22 (47.8)		35 (47.3)	10 (52.6)	
**1**		7 (14.9)	6 (13)		12 (16.2)	1 (5.3)	
**2**		15 (31.9)	17 (37.0)		25 (33.8)	7 (36.8)	
**3**		2 (4.3)	1 (2.2)		2 (2.7)	1 (5.3)	
**Tumor bed surface area, median [Q1, Q3]**	1.1	0.00 [0.00, 135.00]	0.68 [0.00, 147.50]	0.883	1.62 [0.00, 153.00]	0.00 [0.00, 86.00]	0.622
**Tumor cellularity median, [Q1, Q3]**	2.1	0.00 [0.00, 35.00]	1.00 [0.00, 20.00]	0.827	0.50 [0.00, 30.00]	0.00 [0.00, 20.00]	0.543
**Histological lymph node involvement (%)**	0						
**No**		42 (87.5)	41 (89.1)	1.000	67 (89.3)	16 (84.2)	0.825
**Yes**		6 (12.5)	5 (10.9)		8 (10.7)	3 (15.8)	
**Early metabolic response (%), median [Q1, Q3]**	7.4	−59.75 [−71.75, −37.98]	−58.29 [−71.32, −36.44]	0.684	−58.66 [−70.89, −36.44]	−62.98 [−72.27, −39.91]	0.867
**Dose reduction in chemotherapy, n (%)**	0			0.087			0.637
**No**		38 (79.2)	28 (60.9)		54 (72.0)	12 (63.2)	
**Yes**		10 (20.8)	18 (39.1)		21 (28.0)	7 (36.8)	
**Delayed chemotherapy**	0			0.273			0.11
**No**		26 (54.2)	26 (56.5)		41 (54.7)	11 (57.9)	
**Yes**		22 (45.8)	20 (43.4)		34 (45.3)	8 (42.1)	
**36-month EFS (%, 95CI)**	0	82% (70%, 95%)	86% (77%, 97%)	0.853	82% (73%, 92%)	94% (84%, 100%)	0.222

For continuous variables, mean/SD or median/interquartile range were reported based on the normality assumption (Shapiro–Wilk test). Categorical variables were presented as absolute values and proportions. Patient group differences were assessed using either the *t*-test or Kruskal–Wallis test for normally or non-normally distributed continuous variables respectively, and by Pearson’s chi2 test or Fisher’s exact test for categorical variables. The Wilcoxon rank test was used for ordered categorical variables. Differences in the survival of groups are assessed with a log-rank test. Abbreviations: n, number of patients; RCB, residual tumor burden.

**Table 4:** Association between paclitaxel ToDA and outcomes.

Chemotherapy Regimen		Paclitaxel					
	Missing (%)	Median Infusion Time (12:55 pm)			Optimizing Cut-Off by Maximizing Differences in RCB Classes (14:48)		
		Early	Late	*p*-Value	Early	Late	*p*-Value
**Number of patients**		47	46		74	19	
**RCB (class), n (%)**	1.1			0.878			0.631
**0**		22 (46.8)	23 (51.1)		35 (47.3)	10 (55.6)	
**1**		7 (14.9)	6 (13.3)		12 (16.2)	1 (5.6)	
**2**		17 (36.2)	14 (31.1)		25 (33.8)	6 (33.3)	
**3**		1 (2.1)	2 (4.4)		2 (2.7)	1 (5.6)	
**Tumor bed surface area, median [Q1,Q3]**	1.1	0.25 [0.00, 71.00]	0.00 [0.00, 156.00]	0.655	1.62 [0.00, 120.50]	0.00 [0.00, 217.50]	0.992
**Tumor cellularity median, [Q1, Q3]**	2.1	0.00 [0.00, 35.00]	0.00 [0.00, 20.00]	0.732	0.50 [0.00, 30.00]	0.00 [0.00, 5.00]	0.383
**Histological lymph node involvement (%)**	0						
**No**		42 (89.4)	40 (87.0)	0.970	68 (91.9)	14 (73.7)	0.073
**Yes**		5 (10.6)	6 (13.0)		6 (8.1)	5 (26.3)	
**Early metabolic response (%), median [Q1, Q3]**	7.4	−59.36 [−70.89, −38.31]	−63.52 [−73.14, −36.44]	0.856	−59.09 [−70.89, −37.65]	−67.11 [−73.14, −37.50]	0.741
**Dose reduction in chemotherapy, n (%)**	0			0.601			1.000
**No**		35 (74.5)	31 (67.4)		52 (70.3)	14 (73.7)	
**Yes**		12 (25.5)	15 (32.6)		22 (29.7)	5 (26.3)	
**Delayed chemotherapy**	0			0.264			0.758
**No**		29 (61.7)	22 (47.8)		42 (56.8)	9 (47.4)	
**Yes**		18 (38.3)	24 (52.2)		32 (43.2)	10 (52.6)	
**36-month EFS (%, 95CI)**	0	87% (76%, 98%)	83% (73%, 96%)	0.449	83% (74%, 93%)	94% (84%, 100%)	0.254

For continuous variables, mean/SD or median/interquartile range were reported based on the normality assumption (Shapiro–Wilk test). Categorical variables were presented as absolute values and proportions. Patient group differences were assessed using either the *t*-test or Kruskal–Wallis test for normally or non-normally distributed continuous variables respectively, and by Pearson’s chi2 test or Fisher’s exact test for categorical variables. The Wilcoxon rank test was used for ordered categorical variables. Differences in the survival of groups are assessed with a log-rank test. Abbreviations: n, number of patients; RCB, residual tumor burden.

## Data Availability

The data that support the findings of this study are available on request from the corresponding author upon reasonable request.

## Supplementary Materials

The following supporting information can be downloaded at: https://www.mdpi.com/article/10.3390/cancers18081299/s1, Figure S1: Variation of RCB elements according to SIM infusion timing; Figure S2: Cosinor analysis of the residual cancer burden components according to SIM median infusion timing; Figure S3: Association between Paclitaxel ToDA and treatment outcomes, using the EFS-optimized early-late cut-off; Figure S4: Cosinor analysis of the residual cancer burden components according to paclitaxel median infusion timing; Table S1: Treatment characteristics in the overall population; Table S2: Clinical and Tumor characteristics according to ToDA of SIM infusion; Table S3: Association between SIM ToDA and outcomes using optimized cut off maximizing differences in EFS; Table S4: Clinical and Tumor characteristics according to ToDA of paclitaxel infusion; Table S5: Association between paclitaxel ToDA and outcomes using optimized cut off maximizing differences in EFS.

## Abbreviations

ASCO	American Society of Clinical Oncology
BMI	body mass index
BRCA	breast cancer susceptibility gene
DD-DI	dose-dense dose-intense
EFS	event-free survival
ER	estrogen receptor
GTEx	Genotype-Tissue Expression
HER2	human epidermal growth factor receptor 2
HR	hormone receptor
HR	Hazard ratio
NAC	neoadjuvant chemotherapy
OS	overall survival
pCR	pathological complete response
PET-scan	positron emission tomography scan
PR	progesterone receptor
Q2w	every 2 weeks
Qw	every week
RCB	residual cancer burden
RMST	restricted mean survival time
SD	standard deviation
SUVmax	maximum standardized uptake value
ToDA	time of day administration
ToDMR	time of day maximum range
TNBC	triple-negative breast cancer
ZT	zeitgeber time
