# Supplementary material for: Impact of Neoadjuvant Chemotherapy Administration Time of Day on Pathological Response in Patients with Early Triple-Negative Breast Cancer
Source: Cancers (Basel). 2026 Apr 20;18(8):1299. doi: 10.3390/cancers18081299 (PMC13114987; doi:10.3390/cancers18081299)
Supplement: Supplementary file 1 [file cancers-18-01299-s001.zip › Supplementary_Figure S2_revised.pdf]

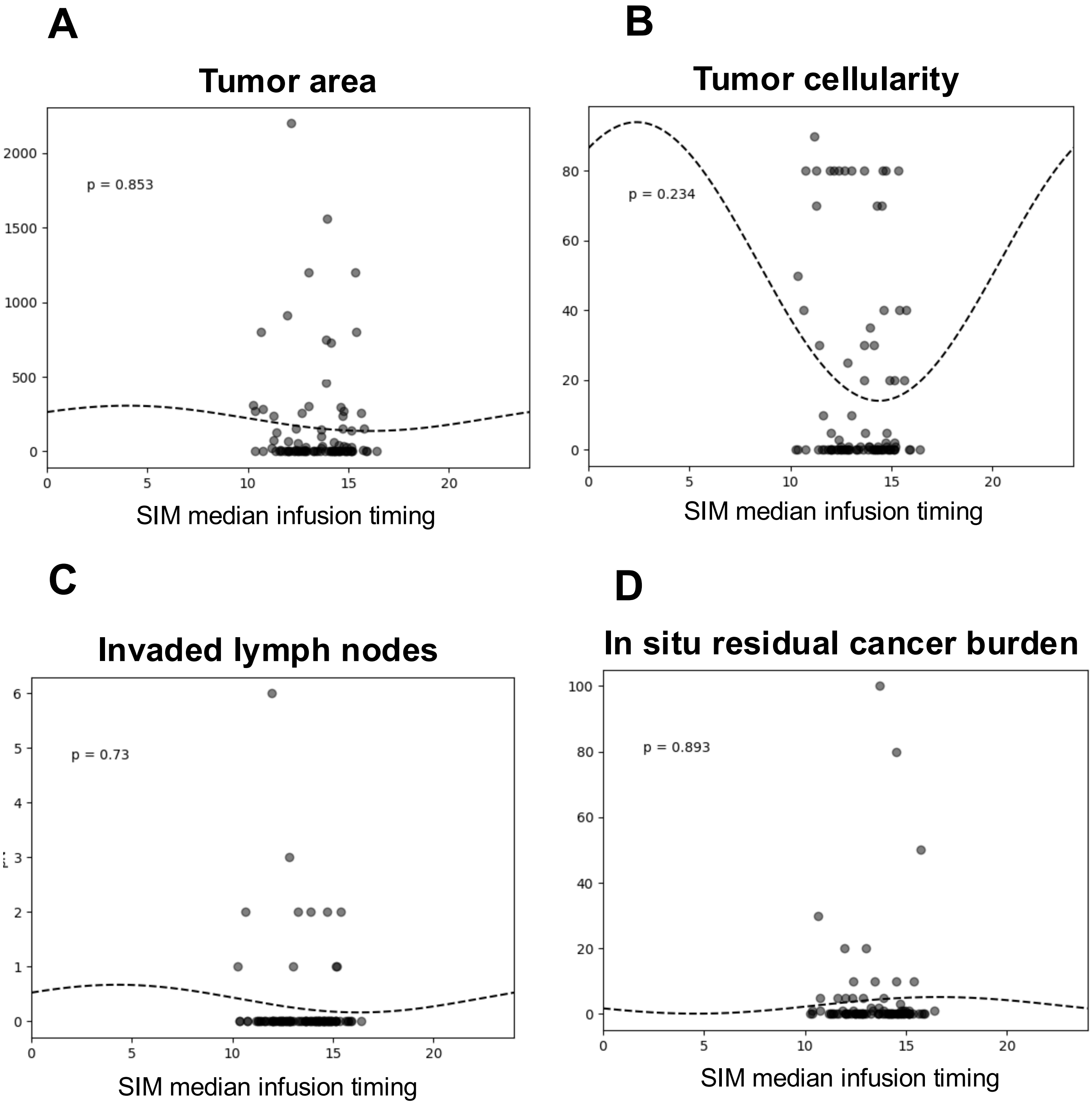

Supplementary Figure S2. Cosinor analysis of the residual cancer burden components according to SIM median infusion timing. Each dot represents the data for an individual patient. P-values are all non-significant here and best-fit 24h-cosine curves are provided as dotted line for indication.
