# Supplementary material for: Impact of Neoadjuvant Chemotherapy Administration Time of Day on Pathological Response in Patients with Early Triple-Negative Breast Cancer
Source: Cancers (Basel). 2026 Apr 20;18(8):1299. doi: 10.3390/cancers18081299 (PMC13114987; doi:10.3390/cancers18081299)
Supplement: Supplementary file 1 [file cancers-18-01299-s001.zip › Supplementary_Figure S3_revised.pdf]

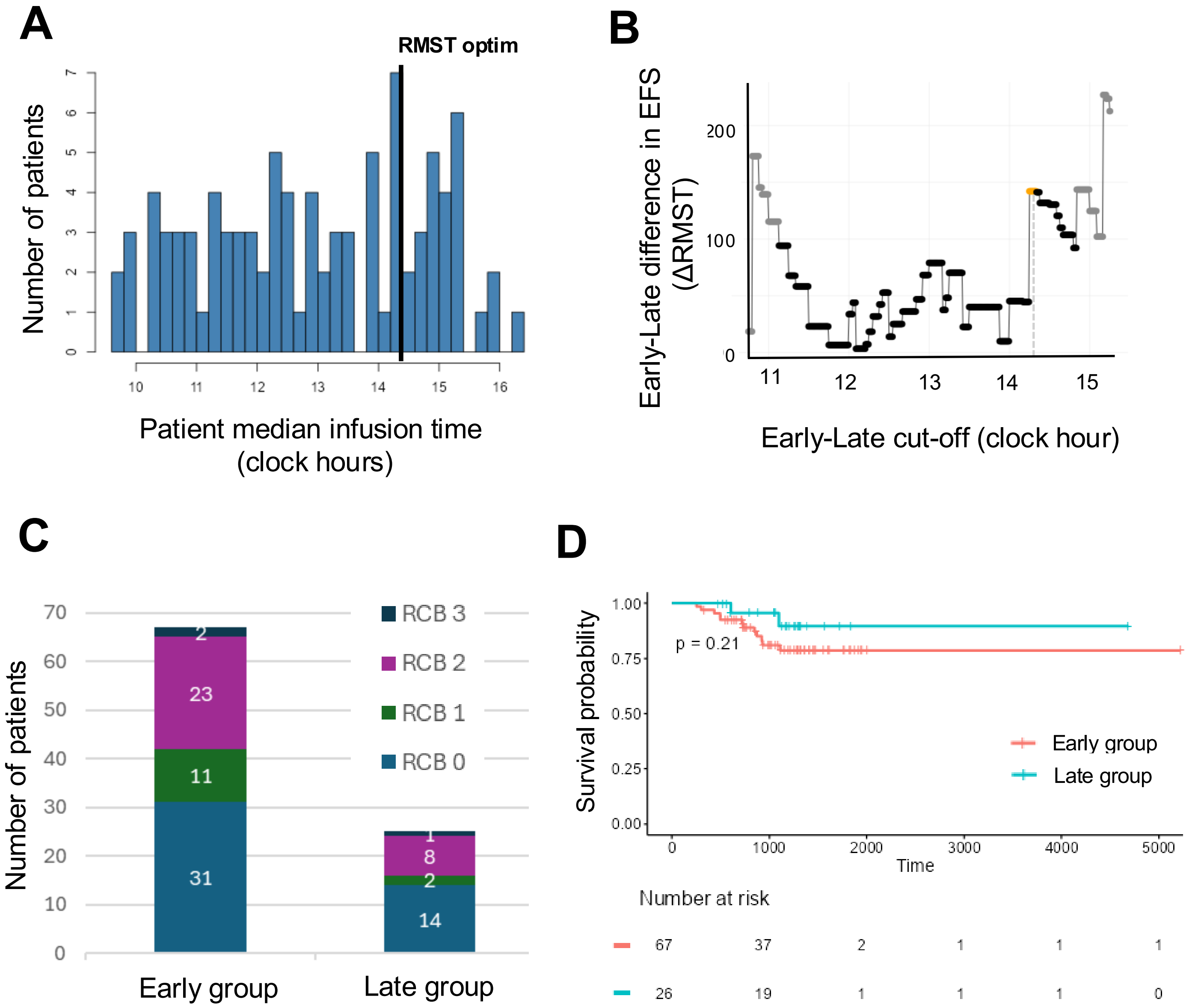

Supplementary Figure S3. Association between Paclitaxel ToDA and treatment outcomes, using the EFS-optimized early-late cut-off. A. Distribution of patient median SIM infusion timing. The black line indicates the timing cut-off maximizing the early-late difference in EFS (ΔRMST, see Methods). **B**. Early/late difference in EFS (ΔRMST) with respect to tested timing cut-off values. The yellow dot indicates the optimal cut-off value corresponding to the maximum difference under group size restrictions. **C** Number of patients of the early and late groups in each RCB class using the EFS-optimized cut-off for defining patient groups. D. Kaplan Meyer curves of EFS using the EFS-optimized cut-off for defining patient groups. P values refer to log-rank test.
